# Correction: Relevance of COVID-19 vaccine on the tourism industry: Evidence from China

**DOI:** 10.1371/journal.pone.0319203

**Published:** 2025-02-06

**Authors:** Fredrick Oteng Agyeman, Zhiqiang Ma, Mingxing Li, Agyemang Kwasi Sampene, Israel Adikah, Malcom Frimpong Dapaah

In the Introduction section, the third sentence of the fifth paragraph was removed.

In the Policy recommendations subsection of Conclusion, there is an error in the second sentence of the fifth paragraph. The correct sentence is: It is believed that these are yet the most simple but effective and efficient ways to curb the spread of the Novel coronavirus.

There is an error in the equation number two. Please view the complete, correct equation here:

$$y=117673.01+123498.62-193.76x_{1}-59.69x_{2}$$
