# Supplementary material for: Relevance of COVID-19 vaccine on the tourism industry: Evidence from China
Source: PLoS One. 2022 Aug 24;17(8):e0269200. doi: 10.1371/journal.pone.0269200 (PMC9401110; doi:10.1371/journal.pone.0269200)
Supplement: S1 File — (DOCX) [file pone.0269200.s001.docx]

**Relevance of COVID-19 vaccine on the tourism industry: Evidence from China**

Supplementary information

S1 Table. Australia’s Total International Tourism Outbound from 2008 - 2020.

| Years | China | Indonesia | New Zealand | Thailand | United Kingdom | United State | Australia |
| --- | --- | --- | --- | --- | --- | --- | --- |
| 2008 | 280,800 | 324,900 | 928,100 | 404,300 | 466,600 | 510,900 | 5,716,200 |
| 2009 | 256,000 | 440,200 | 955,300 | 380,600 | 464,700 | 518,700 | 5,858,200 |
| 2010 | 286,600 | 647,100 | 1,052,800 | 425,700 | 473,700 | 634,800 | 6,680,900 |
| 2011 | 354,600 | 807,100 | 1,060,100 | 486,600 | 496,400 | 763,900 | 7,424,500 |
| 2012 | 369,200 | 922,400 | 1,103,100 | 588,800 | 532,800 | 842,800 | 8,015,600 |
| 2013 | 380,300 | 914,200 | 1,126,300 | 628,200 | 526,500 | 921,000 | 8,401,200 |
| 2014 | 398,300 | 1,015,500 | 1,185,600 | 615,700 | 564,300 | 970,200 | 8,986,700 |
| 2015 | 405,200 | 1,125,600 | 1,224,800 | 564,700 | 570,900 | 999,400 | 9,262,500 |
| 2016 | 447,300 | 1,147,500 | 1,292,600 | 558,200 | 600,600 | 1,061,500 | 9,665,300 |
| 2017 | 505,700 | 1,222,900 | 1,411,600 | 558,200 | 610,900 | 1,086,200 | 10,297,200 |
| 2018 | 572,200 | 1,210,800 | 1,419,000 | 581,400 | 643,700 | 1,084,200 | 10,759,400 |
| 2019 | 610,900 | 1,310,800 | 1,444,200 | 565,300 | 667,800 | 1,078,800 | 11,231,800 |
| 2020 | 396,900 | 1,076,700 | 1,145,000 | 383,000 | 528,000 | 778,700 | 8,559,200 |

Source: [77]


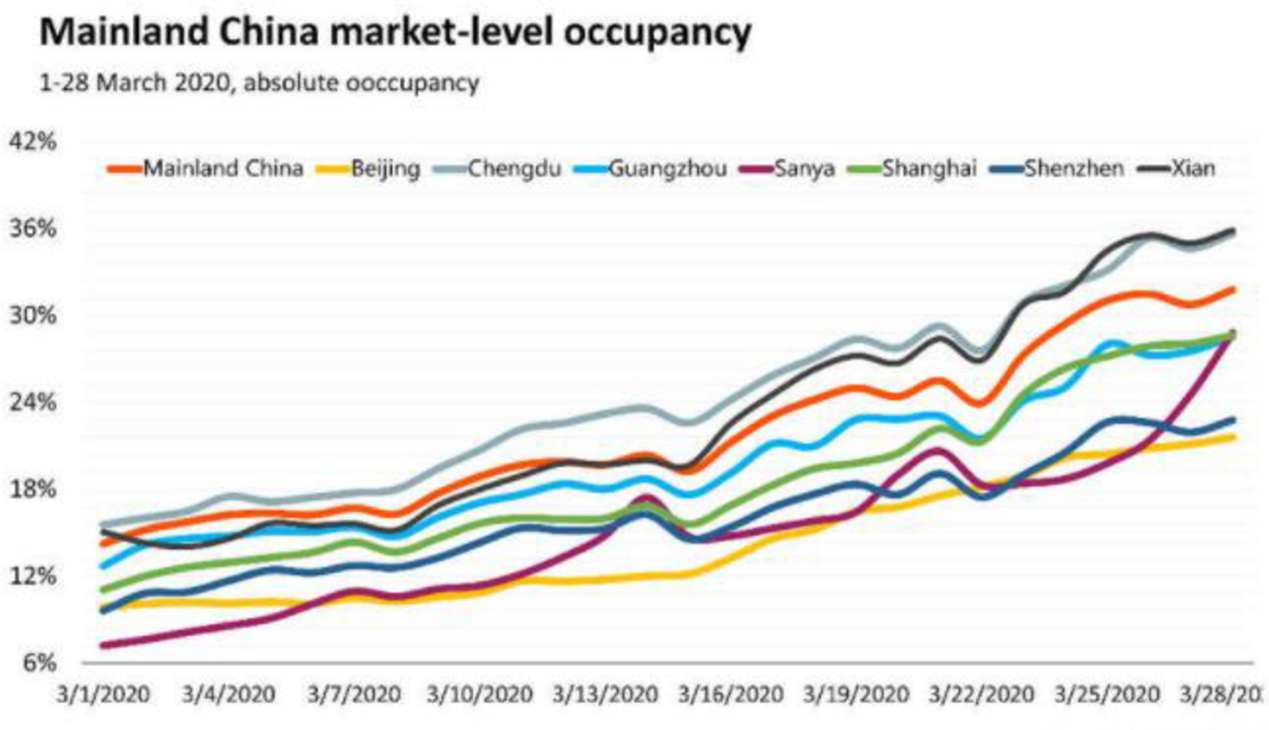


S1 Fig. Mainland China hotel industry shows early signs of performance recovery

Source: [70]


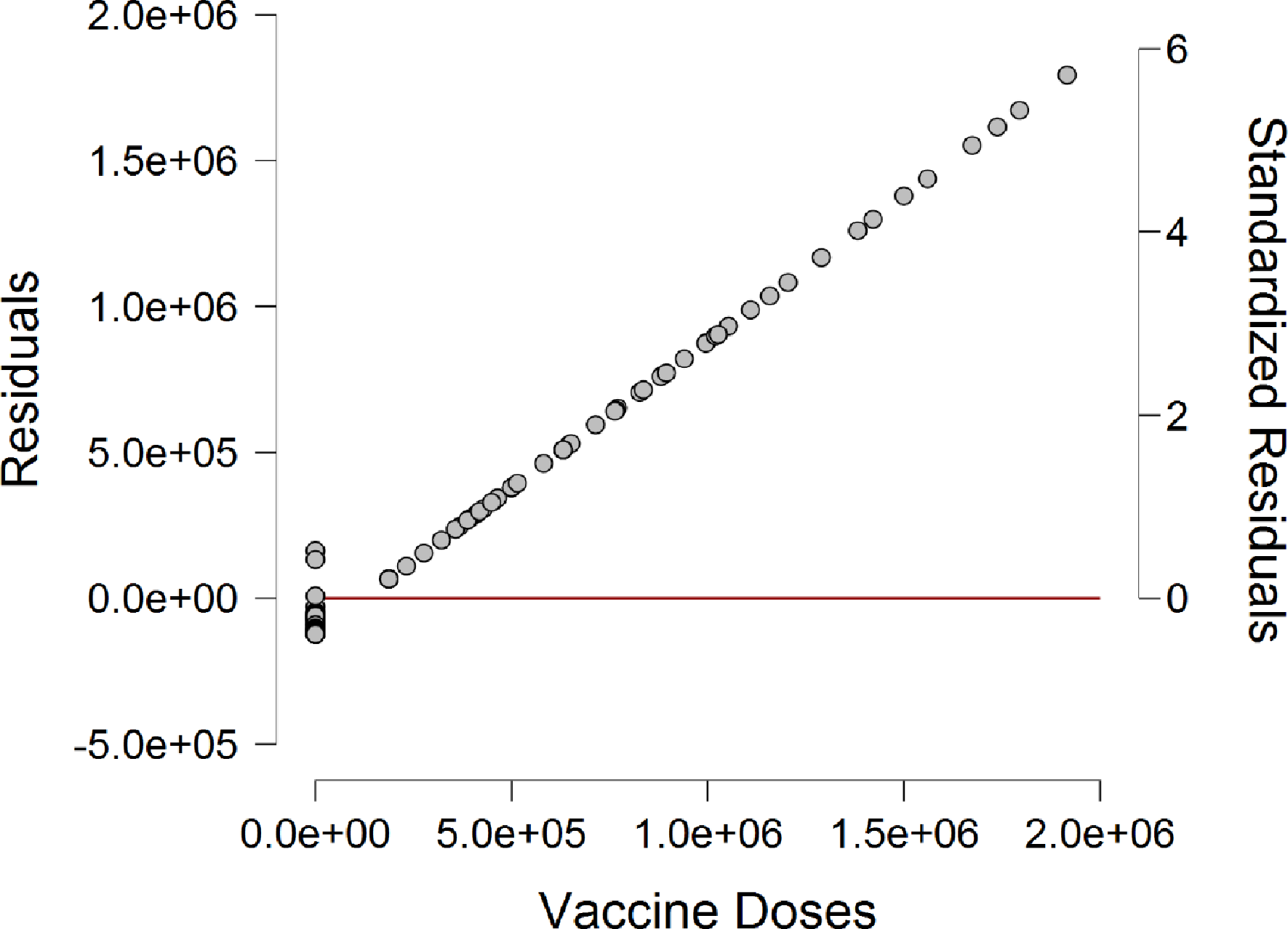


S2 Fig. Residual versus covariate plot

Source: Authors estimation (JASP Software)


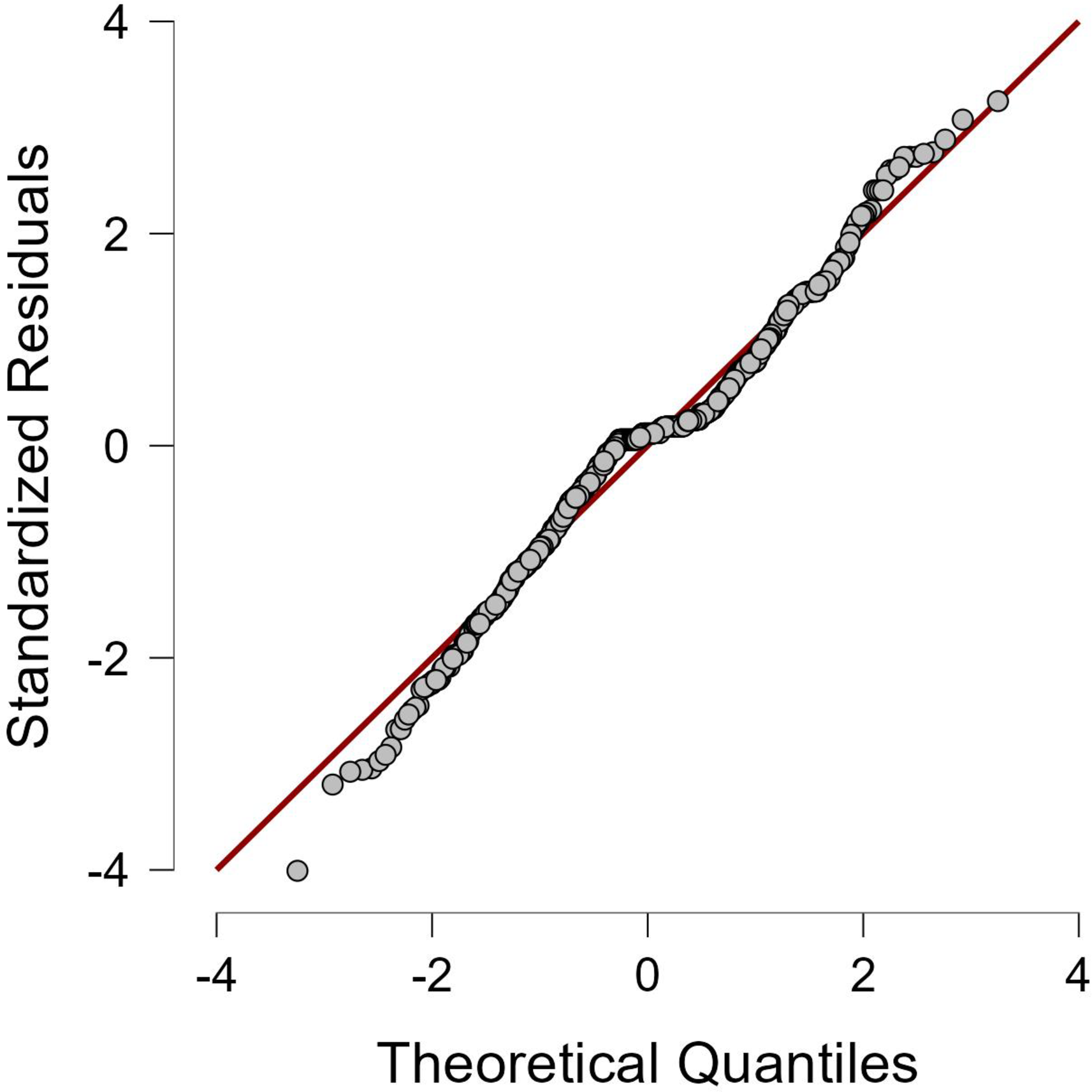


S3 Fig Q-Q Plot Standardized Residuals

Source: Authors estimation (JASP Software)


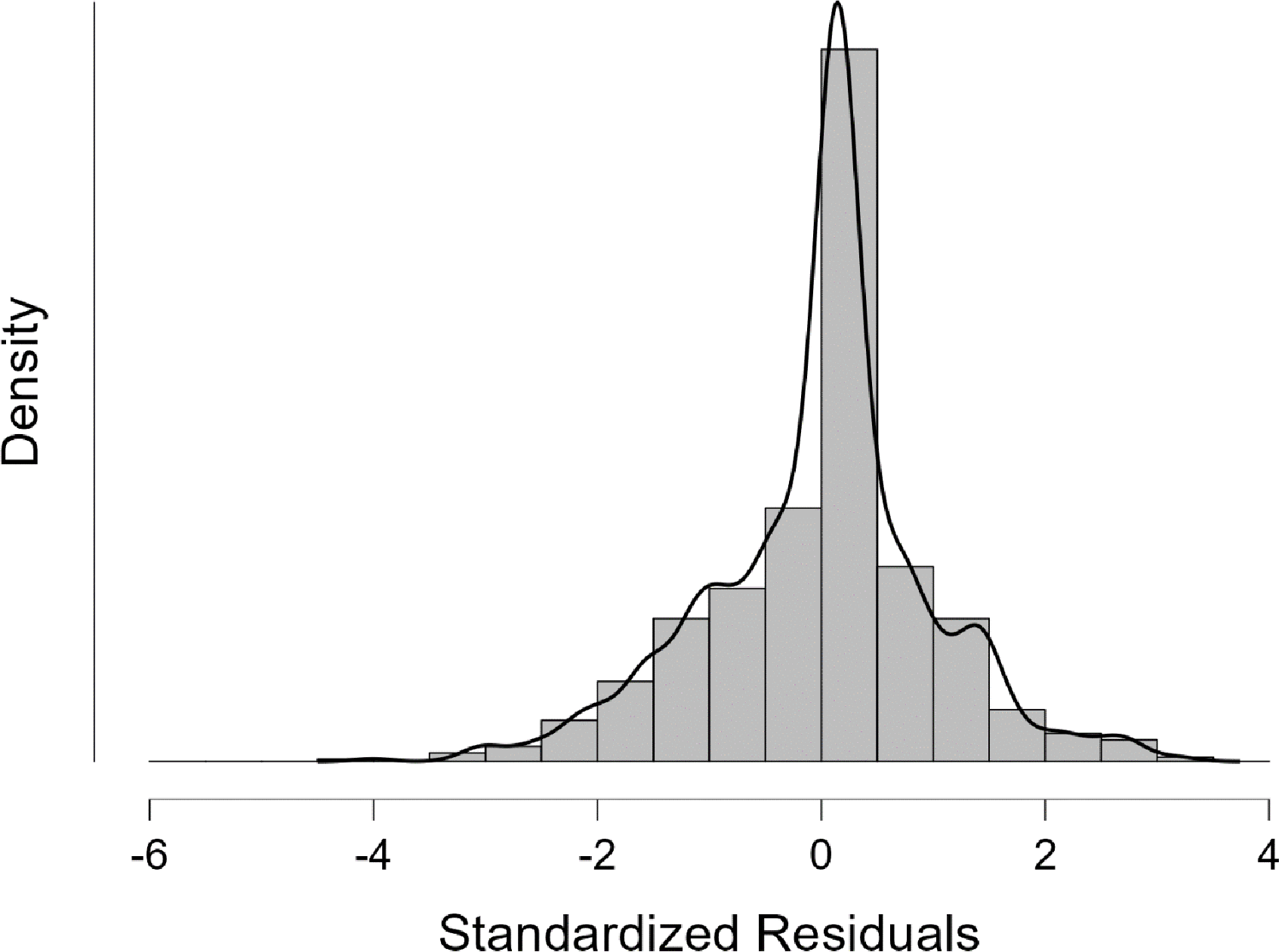


S4 Fig Standardized residual histogram

Source: Authors estimation (JASP Software)
