# Supplementary material for: Relevance of COVID-19 vaccine on the tourism industry: Evidence from China
Source: PLoS One. 2022 Aug 24;17(8):e0269200. doi: 10.1371/journal.pone.0269200 (PMC9401110; doi:10.1371/journal.pone.0269200)
Supplement: Attachment — Submitted filename: Relevance of the COVID-19 vaccine on the Tourism IndustryEvidence from CHINA.pdf [file pone.0269200.s002.pdf]

THE RELEVANCE OF THE COVID-19 VACCINE ON THE TOURISM INDUSTRY: EVIDENCE FROM CHINA

The title should read: Relevance of COVID-19 vaccine on the Tourism Industry: Evidence from China

The Key words should read: COVID-19 vaccine, Tourism Industry, infection, new cases; new death, China

Methodology Section

This section seems interesting as the flow of its introduction rolls out well.

Results and Discussion

Virtually all your tables lack appropriate specific titles.

For example, Table 1: Descriptive Statistics on what?

Table 3: ANOVA of what;

Table 4: Coefficients of what?

Still on the Results and Discussion section, the subsections/subtopics are not properly captured. For instance, "the pattern of Vaccine doses and impact on Death cases and New reported cases in China' need to be recast.

More so, there is poor coherence between your results and discussion section

More so, majority of the tables conventionally lacks a source at the foot of the table

Can the figures mentioned in the results and discussion section be restored from beyond the references section?

Note that the first column in Table 1 should have a title which is suggested to be Measure

Your references are not bad, but there is room to recheck if each of them aligns with the accepted referencing style. More so, the researchers should ensure that all the citations are captured in the reference section.

The manuscript paper is accepted with major corrections.
